# The Unfolding Situation of Caecal Volvulus: A Retrospective Analysis of 36 Cases From a Single Center

**DOI:** 10.7759/cureus.21071

**Published:** 2022-01-10

**Authors:** Vimaladhithan Mahendran, Bala Reddy, Ihab Jaradat

**Affiliations:** 1 General Surgery, Western General Hospital, Edinburgh, GBR; 2 Department of Colorectal Surgery, Worcestershire Acute Hospitals NHS Trust, Worcester, GBR; 3 Department of Colorectal Surgery, NHS Highland, Inverness, GBR

**Keywords:** geriatric emergency, intra-abdominal adhesions, right hemicolectomy, bowel obstruction, caecal bascule, caecal volvulus

## Abstract

Background

Caecal volvulus (CV) is a rare cause of bowel obstruction. However, there has been a steady rise in the number of cases over the decades. The demographic profile of patients developing CV has changed to a much older population. We conducted a retrospective review to determine the incidence, demographic profile, management, and outcomes of CV patients in our institution during the last nine years.

Methodology

A retrospective audit of all patients diagnosed with CV at Worcestershire Acute Hospitals NHS Trust between 01 January 2011 and 31 March 2020 was performed. Patients admitted with any other type of volvuli such as sigmoid volvulus, small bowel volvulus, and gastric volvulus were excluded.

A systematic search of the electronic medical records for all patients admitted under the International Classification of Diseases, Tenth Revision code K562: volvulus was performed for the study duration. It yielded a total of 1,019 patients. After excluding all patients who did not have either a CV or caecal bascule, we included 36 patients in the final analysis.

Results

Most of our patients were females (78%) with a median age was 76 years. The majority (86%) had at least one medical comorbidity, and 36% had a previous abdominal operation. Abdominal pain was the main complaint in 94% of patients. All patients had undergone a computed tomography (CT) scan to confirm their diagnosis. Most of our patients (84%) underwent surgery. Open right hemicolectomy was the most commonly performed operation (87%). Out of the six patients who did not undergo surgery, three responded to bowel rest and nasogastric tube decompression; one patient underwent successful colonoscopic decompression. In contrast, two patients, unfortunately, passed away. The median length of hospital stay was nine days, with a 30-day mortality of 3%.

Conclusions

CV remains a rare cause of bowel obstruction. Most of our patients were old, frail, and had medical comorbidities. More than one-third of patients had undergone previous abdominal surgery. Early CT scan followed by right hemicolectomy was associated with low mortality.

## Introduction

Volvulus of the colon is the third most common cause of colonic obstruction [1]. Initially, caecal volvulus (CV) was considered a rare diagnosis, which occurred predominantly in young males. Over the decades, however, there has been a steady rise in the incidence of CV. The demographic profile has shifted towards a much older population, mainly affecting females [2]. There are several case reports of CV after numerous types of surgeries, ranging from laparoscopic cholecystectomies to cardiac surgery [3-7]. Appendicectomy and gynaecological surgeries have also been identified as common procedures that can lead to CV later in life [7,8]. The lifetime risk of developing acute appendicitis is estimated to be 7-8% worldwide, making it one of the most common general surgical emergencies [9]. The rising incidence of CV in patients who have undergone previous abdominal surgeries may represent long-term morbidity related to their operations.

We aimed to analyse the incidence, demographic profile, comorbidities, surgical management, and outcomes of patients who presented with CV to our institution in the last nine years. Through our review, we hope to raise awareness regarding the changing demographic profile of CV. A high index of suspicion is required to make a prompt diagnosis, and timely surgical intervention is needed to reduce morbidity and mortality.

## Materials and methods

We conducted a retrospective analysis of all patients who were diagnosed with CV between 01 January 2011 to 31 March 2020. Data were collected from the electronic medical records (EMT). This study was conducted at Worcestershire Acute Hospitals NHS Trust, comprising two main hospitals: the Worcestershire Royal Hospital, Charles Hastings Way, Worcester WR5 1DD, and the Alexandra Hospital, Woodrow Drive, Redditch B98 7UB. Institutional Review Board approval was not needed for this retrospective study. However, this study was registered in the Clinical Audit Tracking System with the registration number 10967.

All patients who had a diagnosis of CV or caecal bascule during the study period were included. Patients admitted with any other type of volvuli such as sigmoid volvulus, small bowel volvulus, and gastric volvulus were excluded. A systematic search of the EMR for all patients admitted under the International Classification of Diseases, Tenth Revision code K562: volvulus was performed for the study duration. The search yielded a total of 1,019 patients. After excluding all patients who did not have either a CV or caecal bascule, we included 36 patients in the final analysis.

## Results

**Figure 1:**
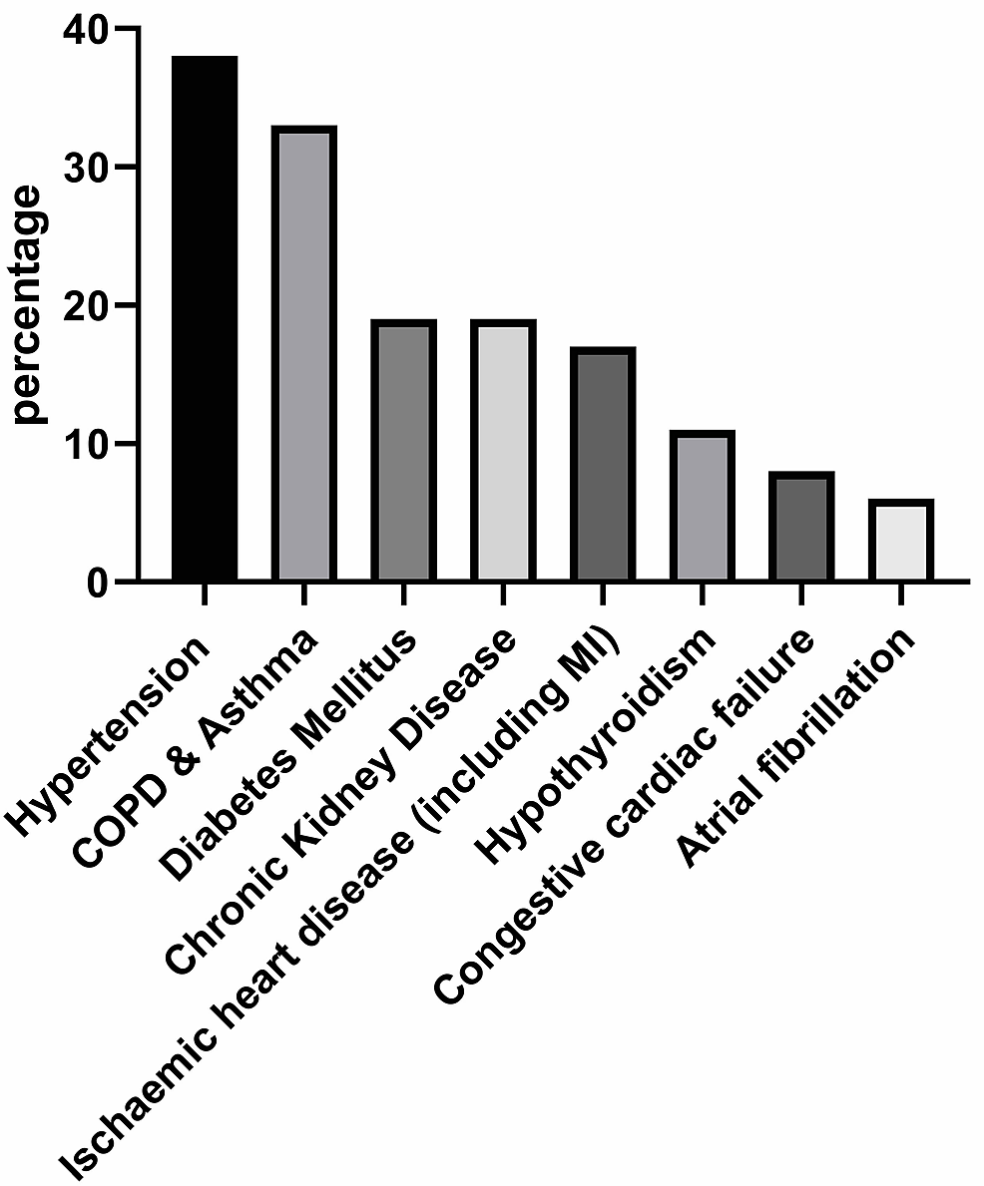
Comorbidities of patients with caecal volvulus. COPD: chronic obstructive pulmonary disease; MI: myocardial infarction

After screening the records of 36 patients, the median age of our patients was 76 (range: 46-89) years. There were 28 (78%) females. All our patients were White British. Most of our patients (86%; n = 31) had at least one medical comorbidity (Figure 1). Hypertension (38%; n = 14) and chronic obstructive pulmonary disease (COPD) (33%; n = 12) were the most common comorbidities. A history of smoking was reported by 39% (n = 14) of our patients. Five (14%) patients reported taking anti-depressant medication at the time of admission.

In this study, the mean body mass index (BMI) was 26 kg/m^2^ (mean = 26, standard deviation [SD] = 5). Obesity, i.e., a BMI of over 30 kg/m^2^, was noted in 22% (n = 8) of our patients. Type 2 diabetes mellitus was prevalent in 19% (n = 7) of our patients. Chronic kidney disease was also noted in 19% (n = 7) of our patients. While 17% (n = 6) of our patients provided a history of ischaemic heart disease, and 6% (n = 2) each had a previous history of stroke and atrial fibrillation. In 11% of patients, a history of hypothyroidism was noted, and 8% (n = 3) suffered from congestive cardiac failure (Figure 1).

More than half of our patients (56%, n = 20) had undergone surgeries in the past. A significant proportion of the patients (36%; n = 13) had undergone previous abdominal operations. Total abdominal hysterectomy (14%, n = 5) was the most common type of surgery (Table 1). All patients underwent a preoperative computed tomography (CT) scan of the abdomen and pelvis.

**Table 1:** Types of previous surgeries.

Type of surgery	Number of patients (%)
Previous abdominal operations	13 (36%)
Patients who have undergone >1 abdominal operations	4 (11%)
Total abdominal hysterectomy	5 (14%)
Emergency laparotomy	3 (8%)
Open appendicectomy	3 (8%)
Open hernia repair (inguinal, incisional, femoral)	3 (8%)
Laparoscopic procedure (adhesiolysis, cholecystectomy, Nissen’s fundoplication)	3 (8%)
Coronary artery bypass	2 (5%)

**Figure 2:**
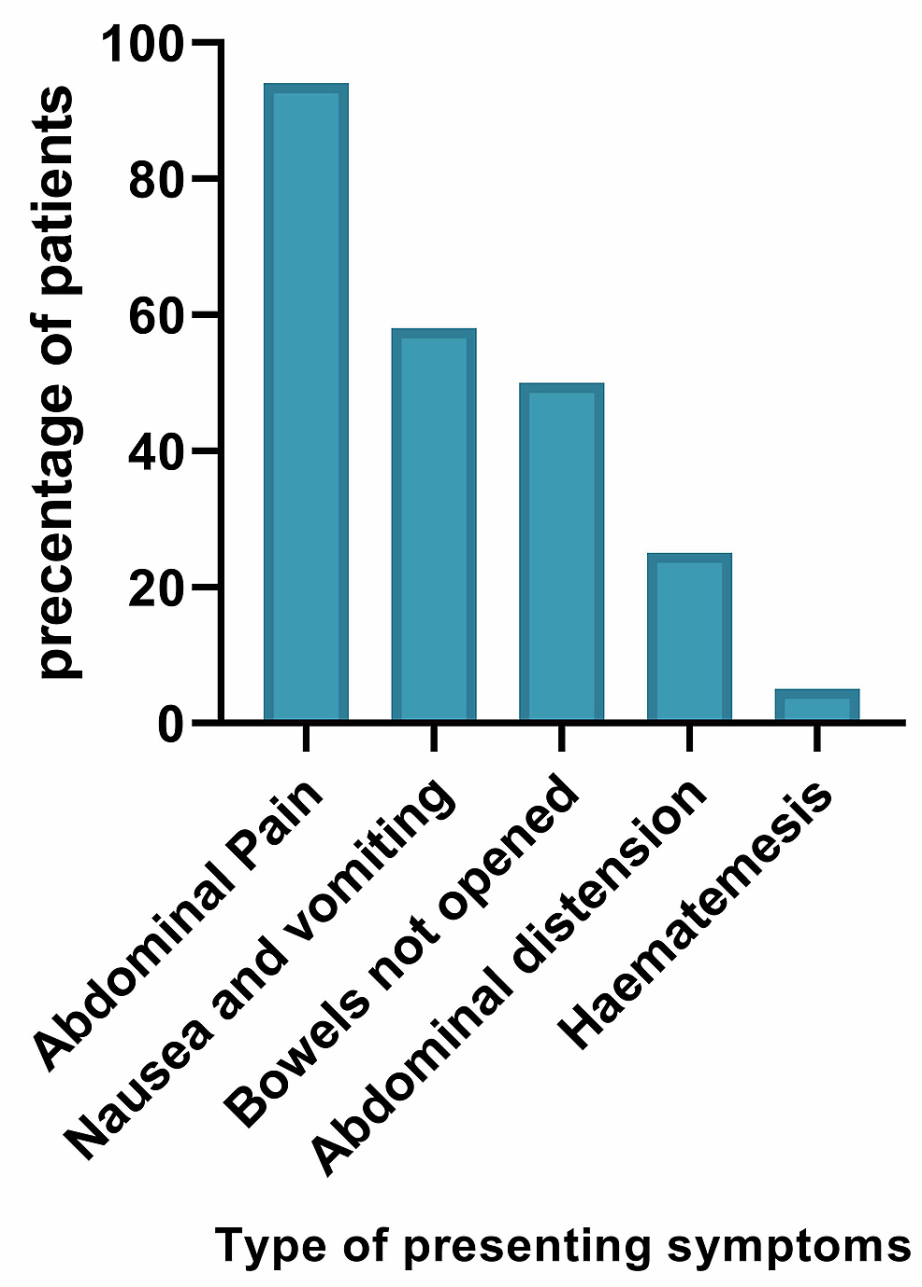
Common presenting symptoms of patients with caecal volvulus.

Figure 2 depicts the various presenting symptoms of patients diagnosed with CV. The most common symptom was abdominal pain (94%), nausea, and vomiting (58%). In 50% (n = 18) of the cases, patients were not passing any flatus, while 25% (n = 9) presented abdominal distension. Haematemesis was the presenting complaint in 5% (n = 2) of our patients. Most (22%, n = 8) patients described their pain to be diffuse or generalised, whereas 16% (n = 6) developed a central abdominal pain (Table 2).

**Table 2:** Site of abdominal pain.

Site of pain	Number of patients (%)
Diffuse or Generalised	8 (22%)
Central	6 (16%)
Right lower abdomen	4 (11%)
Left lower abdomen	3 (8%)
Epigastric	2 (5%)
Data not available	8 (22%)

All patients underwent preoperative CT scans; 28 had a CT diagnosis that matched later intraoperative findings. Other findings of requested CT scans are listed in Table 3.

**Table 3:** Common findings on CT scan. CT: computed tomography

Findings on CT scan	Number of patients (%)
Perforation	4 (11%)
Bowel ischaemia	3 (8%)
Presence of obstructing transverse colon tumour	1 (3%)
Malrotation	1 (3%)

In our series, 84% (n = 30) of the patients had undergone surgery to correct their CV. Open right hemicolectomy was the most commonly performed operation (87%, n = 26). In 33% (n = 12) of cases, CV was uncomplicated. In 30% (n = 9) of cases, the surgeons reported that the caecum had an impending perforation, whereas in 10% (n = 3) of patients, the caecum had perforated. In 17% (n = 5) of patients, the caecal mesentery had torted around an adhesive band. One patient had a mid transverse colon tumour that caused the CV. A primary anastomosis was performed in 80% (n = 24) of our patients at the time of surgery. The most popular choice among our surgeons was stapled side-to-side ileocolic anastomosis (77%, n = 23). See Table 4 for more details of the operative intervention.

**Table 4:** Details of operative intervention. *Information regarding stoma/anastomosis was unavailable for two patients; one patient underwent laparoscopic adhesiolysis.

Types of surgery	Number of patients (%)
Open right hemicolectomy	26 (87%)
Laparoscopy converted to open right hemicolectomy	1 (3%)
Open ileocaecal resection	2 (7%)
Laparoscopic adhesiolysis and appendicectomy	1 (3%)
Intraoperative findings	
Caecal volvulus	12 (33%)
Ischaemia, impending perforation	9 (30%)
Torsion of caecal mesentery around the adhesive band	5 (17%)
Perforated caecum with peritonitis	3 (10%)
Mid transverse colon tumour	1 (3%)
Types of anastomosis*	
Stapled side-to-side ileocolic anastomosis	23 (77%)
Hand-sewn end-to-end anastomosis	1 (3%)
Stomas	
End ileostomy	2 (7%)
Double barrel stoma	1 (3%)
Stapled side-to-side ileocolic anastomosis with loop ileostomy	2 (7%)

The median length of hospital stay was nine (range: 3-53) days. Three patients (8%) required postoperative intensive care unit stay. The overall 30-day mortality of our series was 3% (n = 1). See Table 5 for details of the postoperative complications.

**Table 5:** Postoperative complications. ^#^Concerning patients who underwent re-laparotomy following a right hemicolectomy. The first patient had developed small bowel obstruction due to a knuckle of the small intestine herniating through the lax nylon sutures used to close the abdominal wall. His small intestinal loop was viable during the laparotomy; hence, no resection was required. He recovered well after the re-laparotomy and was subsequently discharged home. The second patient developed septic shock in the ITU two days after undergoing an open right hemicolectomy. On opening her abdomen, the remaining large intestine up to the peritoneal reflection had infarcted. An intraoperative decision was made to abandon and close the abdomen as any further resection was incompatible with life. The patient, unfortunately, passed away the same day. ADH: antidiuretic hormone; TPN: total parenteral nutrition; ITU: intensive therapy unit

Complication	Number of patients (%)
Re-laparotomy^#^	2 (6%)
Intra-abdominal abscess requiring radiological drainage	2 (6%)
Wound infection	2 (6%)
Hospital-acquired pneumonia	2 (6%)
Syndrome of inappropriate ADH secretion	1 (3%)
Central line-associated sepsis	1 (3%)
Bleeding gastric ulcer requiring endoscopy	1 (3%)
Prolonged ileus requiring TPN	2 (6%)

Non-surgical management

Out of the six patients who did not undergo surgery, three were at high risk of morbidity and mortality after surgery due to frailty and associated medical comorbidities. After discussion with the patient and their relatives, a multi-disciplinary team consisting of a surgeon, anaesthetist, and intensivist made a best interest decision to manage these patients conservatively with bowel rest and nasogastric tube decompression instead of operating. These patients had a ward-based ceiling of care and had a Do Not Attempt Cardio-Pulmonary Resuscitation in place. Two of the patients who were started on conservative treatment deteriorated, later started on end-of-life care, and eventually died during the hospital admission. One out of the six patients had successful decompression of the CV with colonoscopy, while the other three responded to conservative management and were subsequently discharged home.

## Discussion

In our series, 78% of patients were females. Historically, CV was commonly reported among men, but studies have reported a steady female preponderance since the 21st century [2]. While earlier CV was considered a disease of the young, the median age in our series was 76 years, with the youngest aged 46 years and the oldest aged 89 years. Our patient population was more elderly compared to the findings from a recent study, which reported the average age to be 57 years [10]. Almost 86% of the patients in our series had at least one medical comorbidity. Hypertension (38%) and COPD or asthma (33%) were the two most common comorbidities. A significant number of patients (39%) smoked regularly, either in the present or past.

The pathogenesis CV is multifactorial. It is interesting to note that autopsy reports have found that 11-25% of adults have sufficient caecal mobility to risk the formation of volvulus [11]. However, the incidence of CV reported worldwide is approximately between 2.8 and 7.1 per million people per year [11]. Hence, there must be other factors that are necessary for patients to develop a CV. At least 36% of our patients had undergone previous abdominal surgeries, with 11% having had more than one operation. Abdominal hysterectomy (14%) followed by emergency laparotomy, open appendicectomy, hernia repair, and laparoscopic procedures (adhesiolysis, cholecystectomy, Nissen’s fundoplication) (8% each) were among the most common operations. None of our patients had undergone laparoscopic appendicectomy in the past; however, 8% had undergone open appendectomies. Given the older age group and predominantly female population in our series, it is not surprising that the most common surgical procedure performed was an abdominal hysterectomy. In other clinical series, between 23% and 70% of patients had undergone previous abdominal surgery. In a recent series published in 2018, authors from the United States reported that 88.2% of their patients had undergone a previous abdominal surgery [2]. Other factors attributed to the occurrence of CV include pregnancy, previous colonoscopy, chronic constipation, a high-fibre diet, and frequent laxative use. It is believed that these factors can predispose susceptible individuals to caecal displacement, hyperperistalsis, and colonic distension resulting in CV [12].

Abdominal pain (94%) was the most common presenting symptom in our patients. Most patients complained that their pain was either generalised or diffuse (22%). Patients with CV may present in three different forms. Chronic intermittent abdominal pain or mobile caecum syndrome is the mildest form of presentation associated with mild right-sided abdominal tenderness. However, half of the patients can progress to acute volvulus [12]. Next, patients may present with cramping abdominal pain along with nausea and vomiting. The pain is progressive, and, on examination, the abdomen may be distended with high pitched bowel sounds [12]. Almost 89% of our patients presented with acute CV. A few patients may present with signs and symptoms of peritonitis and septic shock secondary to perforated CV [12]. This type of presentation is described as acute fulminant colitis, which was seen in 11% of our patients. In our series, all patients underwent a preoperative CT scan of the abdomen and pelvis. Our hospital has round the clock access to CT scans, and the reports are available within a couple of hours. Even though many of the patients had an abdominal radiograph, especially in the emergency department, this was usually inconclusive and did not get reported as quickly as CT scans.

None of our patients underwent barium or gastrografin enemas. Our practise reflects the global shift towards abdominal CT as the initial imaging investigation for acute abdomen [12]. Along with diagnosing CV, CT scans also provide valuable information regarding the vascularity of the bowel, adhesions, or anatomical variations such as malrotation [13]. The three most common CT findings associated with CV are “coffee bean,” “bird beak,” and “whirl sign” [13]. Furthermore, visualisation of a gas-filled appendix and, more recently, ileocolic vasculature curvature have a high sensitivity and specificity for confirming CV on CT scans [13,14]. In our series, four patients were found to have caecal perforation, and three patients had bowel ischaemia. One each was found to have malrotation and an obstructing transverse colon tumour on CT scan. In 78% of the patients, the preoperative diagnosis matched the intraoperative findings.

Most of our patients (84%; n = 30) underwent surgery, while 11% (n = 6) were managed conservatively. In the conservative management group, the mean age was 78 years, and all these patients had significant medical comorbidities, which were associated with a high risk of postoperative mortality and morbidity. All six patients had a CT scan that confirmed CV and excluded bowel ischaemia. Of the six patients, three responded to bowel rest and nasogastric tube decompression and were discharged home. Although it is not our usual practice, one patient had a successful colonoscopic decompression of CV. In two patients, a multi-disciplinary team involving a surgeon, an anaesthetist, and an intensivist found that surgery was associated with a prohibitively high risk of mortality and decided that the ceiling of care must be ward-based. Both patients prioritised their comfort over any active intervention. They did not want any further treatment and wished not to be resuscitated in case of cardiac arrest. When they began to deteriorate clinically, they were started on end-of-life care.

The most common surgical procedure performed was an open limited right hemicolectomy with a stapled ileotransverse anastomosis (72%). One patient underwent a laparoscopic right hemicolectomy, which was converted to open surgery as the caecum was grossly dilated, making visualisation difficult. The caecum was twisted around an adhesive band in another patient, most likely from an earlier hysterectomy. The caecum appeared healthy once it was untwisted after releasing the band; however, the patient’s appendix was inflamed and removed. Because the patient had multiple medical comorbidities and had a poor functional status, to begin with, an intraoperative decision was made not to proceed with a right hemicolectomy. Two patients underwent open ileocaecal resection. During surgery, it was found that the caecum was ischaemic with impending perforation in 30% (n = 9) of patients. In 17% (n = 5) of patients, the caecal mesentery was twisted around an adhesive band. Overall, 10% (n = 3) of patients were found to have four-quadrant peritonitis due to a perforated caecum. One patient had CV secondary to an obstructing mid transverse colon tumour which was picked up on the CT scan.

The median length of stay in hospital in our series was nine (range: 3-53) days. Postoperatively, 37% (n = 11) of patients had grade two or higher complications as per the Clavien-Dindo classification [15]. One patient passed away two days after an open right hemicolectomy from multiple organ dysfunction due to necrosis of the remaining part of her large intestine. Our postoperative morbidity of 37% and mortality of 3% were similar to a 2019 study where the authors analysed the American College of Surgeons National Surgical Quality Improvement database and found overall morbidity and mortality of 45.7% and 3.3%, respectively [16]. According to the authors, age, systemic inflammatory response syndrome, sepsis, septic shock, and American Society of Anesthesiologists class >4 were common risk factors for increased morbidity and mortality [16].

In its 2021 edition of the clinical practice guidelines, the American Society of Colon and Rectal Surgeons (ASCRS) recommends early imaging of suspected volvulus to confirm the diagnosis and expedite care [17]. Abdominal radiographs are only suggestive or diagnostic of CV in 26-42% of cases [17]. The ASCRS guidelines strongly recommend segmental resection in all patients and limiting non-resectional procedures to those patients who are medically unfit to undergo resection [17]. They have found sufficient data to support reducing mortality and leak rate after colectomy and anastomosis for CV [17]. The postoperative mortality in our series was only 3%; this can be attributed to the use of a CT scan to confirm the diagnosis in all cases and early surgery for patients deemed fit.

Our series of 36 patients is one of the largest single-centre studies on CV to date. The findings of our study show a clear shift in the demographic profile towards a much older population. It was interesting to note that more than one-third of patients in our series had a history of smoking and suffered from COPD. Future research in this direction can help us determine if they increase the risk of CV during one’s lifetime. The study demonstrates the advantage of early CT scans and prompt surgical intervention in correcting CV.

The main limitation of our study is that data were collected retrospectively. Hence, there is a risk of data not being recorded at the time of reporting. We have mentioned missing information in the manuscript wherever it was appropriate. The sample is too small to establish causality between risk factors and outcome.

## Conclusions

CV is still a rare cause of large bowel obstruction. Most of our patients were old, frail, and had medical comorbidities. More than one-third of the patients had undergone previous abdominal surgery. An early CT scan helps to confirm the diagnosis. If patients are fit to undergo surgery, proceeding with a right hemicolectomy is associated with low mortality risk.
